# Antiproliferative Effect of *Grammatophyllum speciosum* Ethanolic Extract and Its Bioactive Compound on Human Breast Cancer Cells

**DOI:** 10.1155/2021/3752169

**Published:** 2021-10-04

**Authors:** Verisa Chowjarean, Kamala Sadabpod

**Affiliations:** ^1^Cosmeceutical Research, Development and Testing Center, College of Pharmacy, Rangsit University, Lak Hok, Pathum Thani 12000, Thailand; ^2^Department of Pharmaceutical Technology, College of Pharmacy, Rangsit University, Lak Hok, Pathum Thani 12000, Thailand; ^3^Department of Pharmacy Practice, College of Pharmacy, Rangsit University, Lak Hok, Pathum Thani 12000, Thailand

## Abstract

*Background*/*Aim*. *Grammatophyllum speciosum* Blume exhibits various promising pharmacological activities. However, its effect on breast cancer has not been determined. *Materials and Methods*. The antiproliferation effects of the *G. speciosum* pseudobulb ethanolic extract (GSE) and isovitexin (bioactive constituent) were investigated on the MCF-7 human breast cancer cell line using MTT and colony formation assay. The expression levels of proliferation-regulatory proteins were determined by western blotting. *Results*. Noncytotoxic concentrations of GSE significantly suppressed the proliferation of MCF-7 cells. Tumor colony formation decreased in both number and size. The level of phosphorylated AKT and *β*-catenin was suppressed by GSE treatment. Antiproliferation was observed in isovitexin-treated MCF-7 cells in the form of inhibited colony formation and reduced expression of phosphorylated AKT and *β*-catenin protein. *Conclusions*. This study demonstrates the novel effect of *G. speciosum* as an antiproliferative via suppression of the AKT/*β*-catenin-dependent pathway. This may prompt further investigation of this plant in breast cancer therapy.

## 1. Introduction

Cancer is characterized by uncontrolled growth of abnormal cells and accounts for 9.8 million deaths a year worldwide [1]. Breast cancer causes high morbidity and mortality in women and is the second most common cancer following lung cancer, yet current treatment strategies have not increased the five-year survival rate due to therapy resistance or serious side effects [2]. Therefore, novel effective treatments are needed.

AKT expression is usually increased in breast cancer patients and can cause treatment resistance [3, 4]. In human breast cancer, activation of the phosphatidylinositol 3-kinase (PI3K)/AKT pathway is thought to play a crucial role in tumor cell growth, proliferation, differentiation, migration, and invasion [4]. AKT phosphorylates the downstream signaling protein *β*-catenin, which further increases its transcriptional activity and promotes tumor cell progression, indicating that AKT-dependent regulation of *β*-catenin plays a major role in tumor development [5]. *β*-Catenin is a multifunctional protein that promotes cancer cell proliferation, migration, and survival [6]. Overexpression of *β*-catenin in nuclei/plasma of breast cancer tissue correlates to the histological grade of the tumor [7]. Thus, prevention or suppression of breast cancer proliferation could be achievable through the suppression of AKT/*β*-catenin signaling [8].

**Figure 1:**
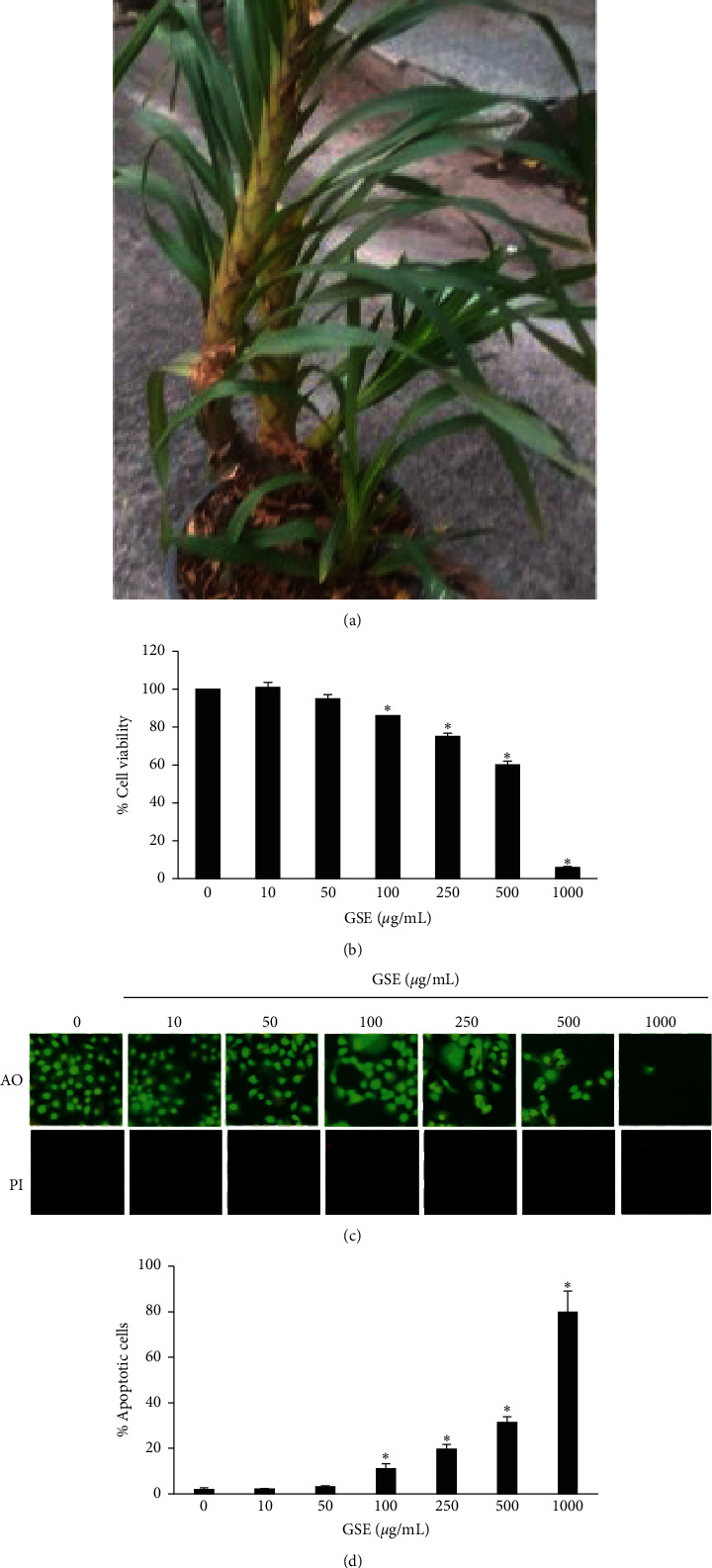
(a) *Grammatophyllum speciosum*. (b) Cell viability of human breast cancer MCF-7 cells. MCF-7 cells were treated with GSE at 0–1000 *μ*g/mL for 24 h, and cell viability was investigated by MTT assay. (c) Level of apoptotic and necrotic cell death was determined by acridine orange/propidium iodine costaining assay after 24 h GSE treatment. (d) Percentages of apoptotic cells were analyzed. Data represent the mean ± SD (*n* = 3). ^*∗*^ Significantly different at *p* < 0.05*versus* nontreated control.

A plant in the Orchidaceae family, *Grammatophyllum speciosum* Blume (Figure 1(a)), is commonly found in Southeast Asia and is used as a pain reliever in traditional medicine. The extract of the pseudobulb of *G. speciosum* is used to relieve pain from scorpion stings and recently reported the antiaging effect [9]. An ethanolic extract of *G. speciosum* pseudobulb (GSE) has been found to protect keratinocytes from apoptosis induced by superoxide anions [10]. That extract has also been found to stimulate wound healing in human skin primal fibroblast cells [11]. The chemical composition of GSE has been analyzed found to include isovitexin, grammatophyllosides, glucosyloxybenzyl derivatives, vandateroside II, cronupapine, vanilloloside, gastodin, and orcinolglucoside [12]. The effects of GSE on human breast cancer have not been assessed. This study examined the anticancer potential of *G. speciosum* and its major compound, isovetexin, on a human breast cancer cell line. The effects on cell proliferation and modulation of the Akt/*β*-catenin signaling pathway were investigated.

## 2. Methods

### 2.1. *Grammatophyllum speciosum* Collection and Extraction

*Grammatophyllum speciosum* Blume pseudobulbs (Figure 1(a)) were harvested from the Khao Hin Sorn Royal Development Study Center, Chachoengsao Province, Thailand, then dried, and granulated. The granules were macerated three times in ethanol (1:9 w/v) at 25°C for three days. The extract was then sieved, and the alcohol evaporated.

### 2.2. Cell Culture

Human breast cancer MCF-7 cell line was obtained from the American Type Culture Collection (Manassas, VA, USA). Cells were cultured in the Roswell Park Memorial Institute (RPMI) 1640 medium supplemented with 10% fetal bovine serum (FBS), 100 U/mL penicillin, 100 *µ*g/mL streptomycin, and 2 mM·L-glutamine. Cells were incubated at 37°C under humid conditions with 5% CO_2_. Cells were subcultured using 0.25% trypsin solution with 0.53 mM EDTA. RPMI 1640 medium, DMEM medium, L-glutamine, FBS, phosphate-buffered saline (PBS), penicillin/streptomycin, and trypsin were obtained from GIBCO (Grand Island, NY, USA). Bovine serum albumin (BSA) and propidium iodide (PI) were obtained from Sigma Chemical (St. Louis, MO, USA). Acridine orange was obtained from Invitrogen (Carlsbad, CA, USA). Antibodies to AKT, phosphorylated AKT, *β*-catenin, and *β*-actin and the secondary antibodies were obtained from Cell Signaling (Danvers, MA, USA).

### 2.3. Cell Viability Assay

The MTT, or 3-(4,5-dimethylthiazol-2-yl)-2,5-diphenyltetrazolium bromide [13], assay was used to determine cell viability after GSE exposure. Cells (1 × 10^4^) were seeded in 96-well plates and treated with GSE (0–1,000 *μ*g/mL). After 24 h incubation, the MTT solution (400 *μ*g/mL) was replaced and incubated at 37°C for 4 h. The MTT solution was removed, and the formazan crystal was dissolved with 100 *μ*L of dimethyl sulfoxide. The optical density of the solution was analyzed using a spectrophotometer (Bio-Rad Laboratories, Hercules, CA, USA) at 570 nm. The data were presented as cell viability (%), calculated by the absorbance of GSE-treated cells relative to that of the control group.

### 2.4. Cell Death Assay

Cells (1 × 10^4^ cells) were seeded in 96-well plates and treated with GSE (0–1,000 *μ*g/mL) for 24 h. Cells were washed with PBS and incubated with 10 *μ*g/mL acridine orange for 30 min and 5 *μ*g/ml PI for 5 min. The DNA fragmentation and nuclear condensation of apoptotic cells and PI-positive necrotic cells were captured under a fluorescence microscope, Olympus CKX41, with an E-330 camera (Olympus, Tokyo, Japan). Apoptotic cells were counted, and % apoptotic cells were calculated by the nuclear condensation cells/total cells at the indicated treatment.

### 2.5. Cell Proliferation Assay

Cells (2 × 10^3^) were seeded in 96-well plates and treated with GSE (0–50 *μ*g/mL) for 72 h at 37°C. Cell proliferation was analyzed by MTT assay, as described above in Section 2.3. Relative proliferative values were calculated by the absorbance of GSE untreated/treated cells at indicated times relative to time 0.

### 2.6. Colony Formation Assay

The colony formation assay was performed to analyze the long-term proliferative abilities of MCF-7 cells. Cells were treated with GSE (0–50 *μ*g/mL) for 14 days in soft agar. Briefly, the bottom layer was prepared using a 1:1 (v/v) mixture of 1% (w/v) agarose and RPMI-1640 medium containing 10% (v/v) FBS. This layer was allowed to solidify for 30 min. The upper cellular layer was a mixture of cells (1 × 10^3^) in a complete medium with 0.3% (w/v) agarose and GSE, which was added onto the bottom layer. Lastly, the complete medium was added on top of the upper layer. Cells were incubated for 14 days at 37°C, and the colony formation was observed and imaged using a phase-contrast microscope (Olympus CKX41 with an E-330 camera).

### 2.7. Western Blot Analysis

Cells (1 × 10^5^) were plated in six-well plates and treated with GSE (0–50 *μ*g/mL) for 48 h. Cells were collected and lysed using lysis buffer (20 mM Tris-HCl [pH 7.5], 10% [v/v] glycerol, 1% [v/v] Triton X-100, 150 mM sodium chloride, 50 mM sodium fluoride, 1 mM sodium orthovanadate, a protease inhibitor cocktail (Roche Molecular Biochemical, Mannheim, Germany), and 100 mM phenylmethylsulfonyl fluoride). The Bio-Rad protein assay kit (Bio-Rad, Hercules, CA, USA) was used to analyze the protein content. Equal amounts of protein from each sample were then separated using SDS polyacrylamide gel electrophoresis. Protein was transferred to 0.45 *μ*m nitrocellulose membranes (Bio-Rad, Hercules, CA, USA), and the blot was blocked by a solution of 5% (w/v) nonfat milk in tris-buffered saline with Tween 20 (TBST) (25 mM Tris-HCl [pH 7.5], 125 mM NaCl, and 0.1% [v/v] Tween 20) for 1 h. Primary antibody was added, and the blot was incubated at 4°C overnight. The membrane was washed three times in TBST and incubated at room temperature for 2 h with horseradish peroxidase- (HRP-) conjugated secondary antibody. An enhancement chemiluminescence substrate (Supersignal West Pico; Pierce, Rockford, IL, USA) was used to detect the protein. The level of protein was analyzed using ImageJ software.

### 2.8. Isovitexin Determination by HPLC

The amount of isovitexin, a major compound of *G. speciosum* extract, was investigated using rapid high-performance liquid chromatography (HPLC) using gradient elution with acetonitrile-water as a mobile phase.

### 2.9. Statistical Analysis

The results from each experiment are presented as mean ± SD. Analysis of variance (ANOVA) was used to compare the significant differences between multiple groups. The individual comparisons at *p* < 0.05 significance level were analyzed using Scheffe's post hoc test.

## 3. Results

### 3.1. Effect of *G. speciosum* on the Viability of Human Breast Cancer MCF-7 Cells

We determined a nontoxic concentration of GSE on viabilities of MCF-7 breast cancer cells. The cells were treated with 0–1,000 *μ*g/mL GSE for 24 h, and the cell viabilities were examined by MTT assay. Cell viability was significantly less in cells treated with GSE concentrations of 100 *μ*g/mL or greater, whereas GSE was nontoxic at lower concentrations (Figure 1(b)).

Cell death induction by GSE was evaluated using acridine orange and the PI staining assay. The condition of the same cultures was examined for 24 h, and the chromatin condensation and nuclear fragmentation of apoptotic cells and necrotic cell death were observed. Neither apoptotic nor necrotic cells were found in 10 and 50 *μ*g/mL GSE treatment cultures. In contrast, cells subjected to the higher GSE concentrations showed a greater number of condensed or fragmented nuclei than the control group (Figures 1(c) and 1(d)). Nontoxic GSE concentrations (10–50 *μ*g/mL) were selected for further investigation.

### 3.2. Effect of *G. speciosum* Extract on MCF-7 Cell Proliferation

**Figure 2:**
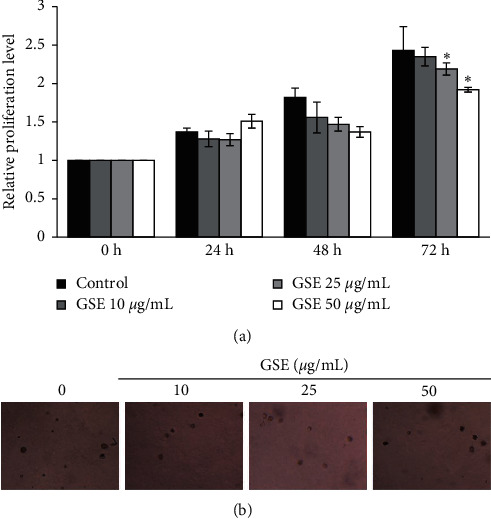
Cell proliferation of MCF-7 cells after exposure to GSE. (a) MCF-7 cells were treated with GSE at 0–50 *μ*g/mL for 72 h, and cell viability was investigated by MTT assay. The relative cell proliferation at 24, 48, and 72 h was calculated compared to time 0. (b) Colony formation was determined by microscopy (4×) after 14 days of GSE treatment. Data represent the mean ± SD (*n* = 3). ^*∗*^Significantly different at *p* < 0.05*versus* nontreated control.

The effect of GSE on the proliferation of MCF-7 cells is shown in Figure 2. MCF-7 cells were cultured with nontoxic concentrations of GSE (10–50 *μ*g/mL) for 72 h and examined for cell proliferation by MTT assay. At 72 h, GSE at 25 and 50 *μ*g/mL caused a significant decrease in the proliferative activity of the cells. There was no significant difference in the percentage of cell proliferation among cells treated with 10 *μ*g/mL GSE (Figure 2(a)). These results further indicate that GSE had an antiproliferative effect against MCF-7 breast cancer cells.

### 3.3. *Grammatophyllum speciosum* Extract Decreases Colony Formation in MCF-7 Cells

Cells were subjected to the colony formation assay to determine the long-term influence of GSE on MCF-7 cell proliferation. Briefly, to prevent cell-to-cell interaction and extracellular matrix adherence, MCF-7 cells were seeded in an agarose layer and treated with GSE at nontoxic concentrations (0–50 *μ*g/mL). The appearance of colonies after 14 days of incubation was imaged as described in Section 2. The results showed that GSE at a concentration of 10–50 *μ*g/mL decreased both the number and size of MCF-7 cell colonies formed (Figure 2(b)). These results demonstrate the antiproliferation ability of GSE toward MCF-7 cells.

### 3.4. GSE Inhibits MCF-7 Cell Growth via Suppression of AKT

**Figure 3:**
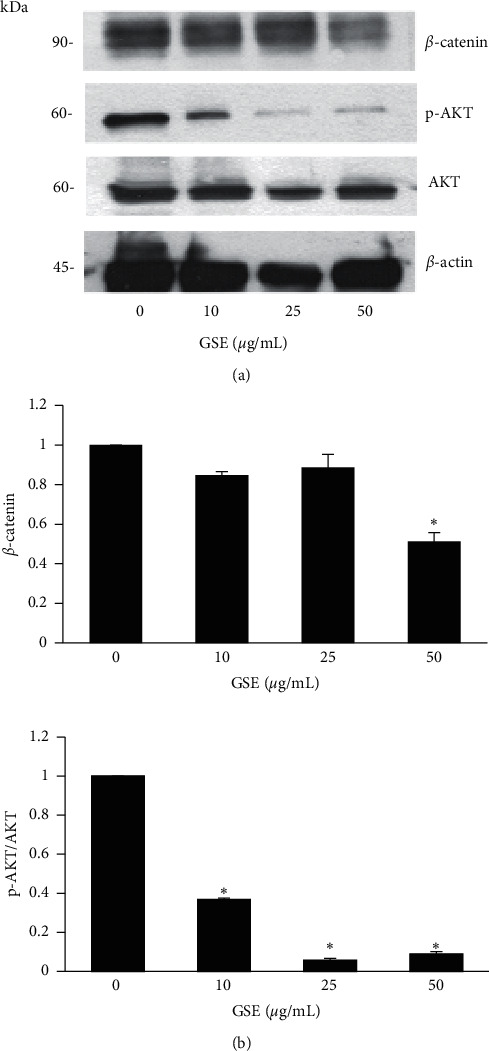
Suppression of survival-associated proteins by GSE. (a) MCF-7 cells were treated with GSE at 0–50 *μ*g/mL for 48 h, and the expression of *β*-catenin, phosphorylated AKT (p-AKT), and total AKT was measured using western blot analysis. (b) Relative protein levels were analyzed by densitometry. Data represent the mean ± SD (*n* = 3). ^*∗*^Significantly different at *p* < 0.05*versus* nontreated control.

It is well known that increasing expression of survival pathways such as AKT causes cell proliferation [14]. This study further investigated whether the inhibition of tumor cell proliferation by GSE was mediated through these survival signals. MCF-7 cells were treated with 0–50 *μ*g/mL GSE, and the intensity of AKT was measured by western blotting. These results reveal significantly less phosphorylated AKT—the active form—in the GSE-treated cells than in the control group (Figures 3(a) and 3(b)). The active AKT is a key signaling protein for the induction of the downstream protein *β*-catenin. In addition, we found that the *β*-catenin protein level decreased after GSE treatment (Figures 3(a) and 3(b)). Taken together, these results indicate that GSE inhibits tumor cell proliferation by suppressing the AKT pathway.

### 3.5. Analysis of Isovitexin in GSE

**Figure 4:**
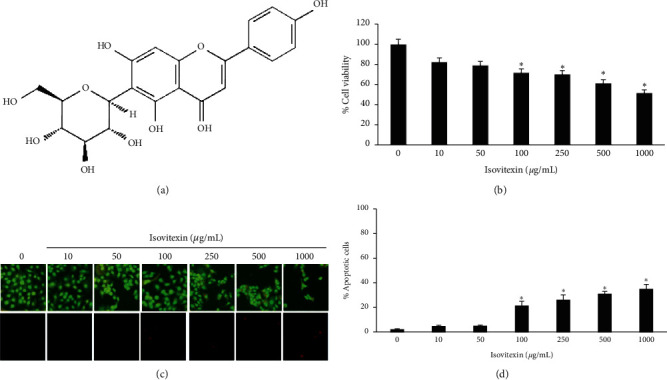
(a) Structure of isovitexin. (b) Cell viability of human breast cancer MCF-7 cells after treated with isovitexin at 0–1000 *μ*g/mL for 24 h. Cell viability was determined by MTT assay. (c) Apoptotic and necrotic cell death were analyzed by acridine orange/propidium iodine costaining assay after 24 h isovitexin treatment. (d) Percentages of apoptotic cells were analyzed. Data represent the mean ± SD (*n* = 3). ^*∗*^Significantly different at *p* < 0.05*versus* nontreated control.

Having shown the cell growth suppressing effect of GSE in human MCF-7 breast cancer cells, we next confirmed that such an effect was exhibit by the major compound. Previous reports showed that GSE contains several phytochemical compounds, including isovitexin (Figure 4(a)) [15]. By HPLC, we found isovitexin was one of the major phytochemical compounds in GSE with a concentration of 0.391 ± 0.012 mg/g.

### 3.6. Cell Growth Inhibition Effect by Isovitexin in MCF-7 Cells

**Figure 5:**
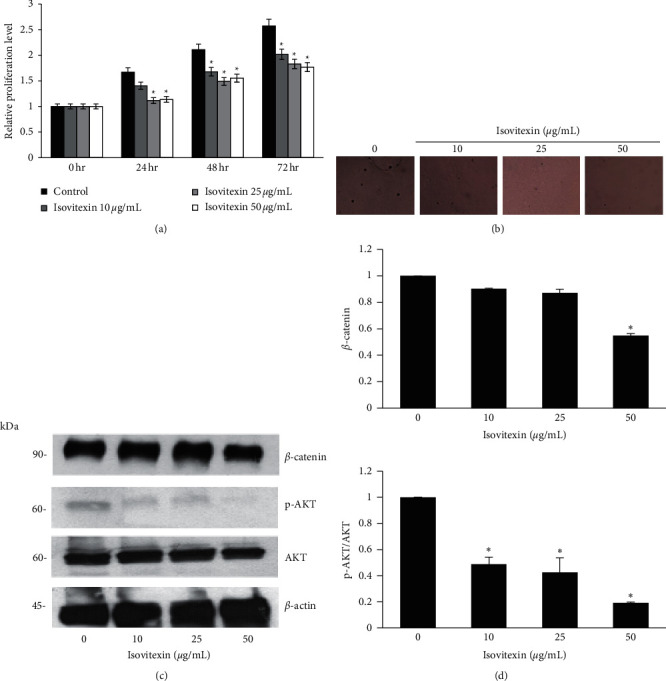
Cell proliferation and survival proteins expression of MCF-7 cells after treated with isovitexin. (a) MCF-7 cells were treated with isovitexin at 0–50 *μ*g/mL for 72 h, and cell viability was determined by MTT assay. The relative cell proliferation at 24, 48, and 72 h was calculated compared to time 0. (b) Colony formation was determined by microscopy (4×) after 14 days of isovitexin treatment. (c) The expression of *β*-catenin, phosphorylated AKT (p-AKT), and total AKT were analyzed by western blot analysis after treated with isovitexin at 0–50 *μ*g/mL for 48 h. (d) Relative protein levels were analyzed by densitometry. Data represent the mean ± SD (*n* = 3). ^*∗*^Significantly different at *p* < 0.05*versus* nontreated control.

Cells were treated with isovitexin to determine the noncytotoxic concentration by MTT assay and acridine orange/PI staining assay. Concentrations of 0–50 *μ*g/mL isovitexin were found not to affect cell viability (Figure 4(b)) and were not observed to condense or fragment cell nuclei (Figures 4(c) and 4(d)). Cell proliferation and colony formation were examined as previously investigated in GSE-treated MCF-7 cells. Treatment of MCF-7 cells with isovitexin for 72 h significantly decreased cell proliferation (Figure 5(a)). The number and size of MCF-7 colonies formed were also decreased by isovitexin (Figure 5(b)). The key survival pathway protein, active AKT, and downstream singling protein *β*-catenin were also significantly reduced (Figures 5(c) and 5(d)). Such observations indicate that isovitexin exhibits an antiproliferative effect in breast cancer cells.

## 4. Discussion

Breast cancer is the most frequently diagnosed cancer in women and one of the major causes of mortality [2]. Effective methods with fewer side effects than conventional treatments should be further investigated. To this end, the anticancer activity of GSE on breast cancer cells has been demonstrated in this study.

Results of the cell proliferation and colony formation assays in this study showed that GSE exerts antiproliferation effects on human breast cancer cells (Figure 2). The PI3K/AKT pathway is one of the most important pathways in human cancer cells [14], and we demonstrated the downregulation of active AKT by GSE treatment (Figure 3). Overactivation of AKT contributes to tumorigenesis, tumor cell proliferation, metastasis, and other cancer activity [7,15]. In our study, GSE suppressed the phosphorylated-AKT protein level, which showed, at a minimum, that this extract has antiproliferation activity against breast cancer cells by inhibiting the AKT pathway.

*β*-Catenin is a multifunctional protein that plays a vital role as a regulatory protein in many cellular processes, including tumor cell survival, proliferation, and metastasis [6]. *β*-Catenin is regulated by the expression of AKT protein via GSK-3*β*. Khramtsov found that cytoplasmic and nuclear accumulation of *β*-catenin is frequently observed in more invasive breast cancers [16]. *β*-Catenin-knockdown breast cancer cells significantly lost the ability to form colonies in the soft agar assay, indicating the role of *β*-catenin in suppressing cell proliferation [17]. GSE suppressing the expression of the *β*-catenin protein (Figure 3) suggests a possible mechanism of GSE in decreasing breast cancer cell proliferation.

Though the antiproliferative effect from crude extract of *G. speciosum* have been evaluated pharmacologically, one of the active constituents, isovitexin, has been evaluated against breast cancer in this study. We found that isovitexin exerts antineoplastic effects on MCF-7 breast cancer cell line by reducing cell proliferation and colony formation (Figure 5). At the molecular level, isovitexin decreased the expression of active AKT and *β*-catenin (Figure 5). In previous research, vitexin exerted antineoplastic effects by reducing cell motility via inhibition of the HIF-1*α* pathway [18].

Isovitexin is lipophilic as its Log *p* value is 0.2 (Log *p* > 0) [19]. It might pass through the cell membrane and exert antioxidation activity [20]. The antioxidant effect of natural lipophilic compounds, such as melatonin, was related to an antiproliferative effect via AKT suppression in C6 glioma cells [21]. In a recent finding, the inactivation of the AKT signaling pathway by isovitexin might be due to its ability to regulate cellular redox state.

## 5. Conclusions

In conclusion, this study demonstrated that GSE has an antiproliferative effect on breast cancer cells by suppressing the AKT/*β*-catenin pathway, possibly due to the bioactivity of isovitexin. The development of *G. speciosum* for breast cancer treatment in combination with standard therapy deserves further study. However, the cellular mechanism of the antiproliferative effect should be further confirmed in the other cell types of the breast cancer cell model and *in vivo* study.

## Data Availability

All data used to support the findings of this study are included within the article.
